# Comparative genomics of human *Lactobacillus crispatus* isolates reveals genes for glycosylation and glycogen degradation: implications for in vivo dominance of the vaginal microbiota

**DOI:** 10.1186/s40168-019-0667-9

**Published:** 2019-03-29

**Authors:** Charlotte van der Veer, Rosanne Y. Hertzberger, Sylvia M. Bruisten, Hanne L. P. Tytgat, Jorne Swanenburg, Alie de Kat Angelino-Bart, Frank Schuren, Douwe Molenaar, Gregor Reid, Henry de Vries, Remco Kort

**Affiliations:** 1 Department of Infectious Diseases, Public Health Service, GGD, Amsterdam, The Netherlands; 2 Department of Molecular Cell Biology, Faculty of Science, O|2 Lab Building, VU University, De Boelelaan 1108, 1081 HZ Amsterdam, The Netherlands; 3 Institute of Microbiology, ETH Zürich, Zurich, Switzerland; 4 Netherlands Organization for Applied Scientific Research (TNO), Microbiology and Systems Biology, Zeist, The Netherlands; 5 Canadian R&D Centre for Human Microbiome and Probiotics, Lawson Health Research Institute, London, Canada; 6 Departments of Microbiology and Immunology, and Surgery, Western University, London, Ontario Canada; 7 Amsterdam Public Health Research Institute, Amsterdam UMC, Amsterdam, The Netherlands; 8 ARTIS-Micropia, Amsterdam, The Netherlands

## Abstract

**Background:**

A vaginal microbiota dominated by lactobacilli (particularly *Lactobacillus crispatus*) is associated with vaginal health, whereas a vaginal microbiota not dominated by lactobacilli is considered dysbiotic. Here we investigated whether *L. crispatus* strains isolated from the vaginal tract of women with *Lactobacillus-*dominated vaginal microbiota (LVM) are pheno- or genotypically distinct from *L. crispatus* strains isolated from vaginal samples with dysbiotic vaginal microbiota (DVM).

**Results:**

We studied 33 *L. crispatus* strains (*n* = 16 from LVM; *n* = 17 from DVM). Comparison of these two groups of strains showed that, although strain differences existed, both groups degraded various carbohydrates, produced similar amounts of organic acids, inhibited *Neisseria gonorrhoeae* growth, and did not produce biofilms. Comparative genomics analyses of 28 strains (*n* = 12 LVM; *n* = 16 DVM) revealed a novel, 3-fragmented glycosyltransferase gene that was more prevalent among strains isolated from DVM. Most *L. crispatus* strains showed growth on glycogen-supplemented growth media. Strains that showed less-efficient (*n* = 6) or no (*n* = 1) growth on glycogen all carried N-terminal deletions (respectively, 29 and 37 amino acid deletions) in a putative pullulanase type I protein.

**Discussion:**

*L. crispatus* strains isolated from LVM were not phenotypically distinct from *L. crispatus* strains isolated from DVM; however, the finding that the latter were more likely to carry a 3-fragmented glycosyltransferase gene may indicate a role for cell surface glycoconjugates, which may shape vaginal microbiota-host interactions. Furthermore, the observation that variation in the pullulanase type I gene is associated with growth on glycogen discourages previous claims that *L. crispatus* cannot directly utilize glycogen.

**Electronic supplementary material:**

The online version of this article (10.1186/s40168-019-0667-9) contains supplementary material, which is available to authorized users.

## Introduction

The vaginal mucosa hosts a community of commensal, symbiotic, and sometimes pathogenic micro-organisms. Increasing evidence has shown that the bacteria within this community, referred to here as the vaginal microbiota (VM), play an important role in protecting the vaginal tract from pathogenic infection, which can have far-reaching effects on a woman’s sexual and reproductive health [1, 2]. Several VM compositions have been described, including VM dominated by (1) *Lactobacillus iners*, (2) *L. crispatus*, (3) *L. gasseri*, (4) *L. jensenii*, and (5) VM that are not dominated by a single bacterial species but rather consist of diverse anaerobic bacteria, including *Gardnerella vaginalis* and members of Lachnospiraceae, Leptotrichiaceae and Prevotellaceae [3–5]. Particularly, VM that are dominated by *L. crispatus* are associated with vaginal health, whereas a VM consisting of diverse anaerobes—commonly referred to as vaginal dysbiosis—has been shown to increase a woman’s odds for developing bacterial vaginosis (BV), acquiring STIs, including HIV, and having an adverse pregnancy outcome [1, 2, 4, 6].

The application of human vaginal *L. crispatus* isolates as therapeutic agents to treat dysbiosis may have much potential [7, 8], but currently, there are still many gaps in our knowledge concerning the importance of specific physiological properties of *L. crispatus* for a sustained domination on the mucosal surface of the vagina. Comparative genomics approaches offer a powerful tool to identify novel important functional properties of bacterial strains. The genomes of nine human *L. crispatus* isolates have previously been studied, also in the context of vaginal dysbiosis [9, 10]. Comparative genomics of these strains showed that about 60% of orthologous groups (genes derived from the same ancestral gene) were conserved among all strains, i.e., comprising a “core” genome [10]. The accessory genome was defined as genes shared by at least two strains, while unique genes are specific to a single strain. Currently, it is unclear whether traits pertaining to in vivo dominance are shared by all strains (core genome) or only by a subset of strains (accessory genome). For example, both women with and without vaginal dysbiosis can be colonized with *L. crispatus* (see, e.g., [11]), and we do not yet fully understand why in some women *L. crispatus* dominates and in others it does not.

The following bacterial traits may be of importance for *L. crispatus* to successfully dominate the vaginal mucosa: (1) the formation of an extracellular matrix (biofilm) on the vaginal mucosal surface, (2) the production of antimicrobials such as lactic acid, bacteriocins, and H_2_O_2_ that inhibit the growth and/or adhesion of urogenital pathogens, (3) efficient utilization of available nutrients—particularly glycogen, as this is the main carbon source in the vaginal lumen, and (4) the modulation of host-immunogenic responses. Considering these points, firstly, Ojala et al. [10] observed genomic islands encoding enzymes involved in exopolysacharide (EPS) biosynthesis in the accessory genome of *L. crispatus* and postulated that strain differences in this trait could contribute to differences in biofilm formation, adhesion, and competitive exclusion of pathogens. Secondly, experiments have shown that *L. crispatus* effectively inhibits urogenital pathogens through lactic acid production, but these studies included only strains originating from healthy women [12–16]. Abdelmaksoud et al. [9] compared *L. crispatus* strains isolated from *Lactobacillus-*dominated VM (LVM) with strains isolated from dysbiotic VM (DVM) and indeed observed decreased lactic acid production in one of the strains isolated from DVM, providing an explanation for its low abundance. However, no significant conclusion could be made as their study included only eight strains. Thirdly, there is a general consensus that vaginal lactobacilli (including *L. crispatus*) ferment glycogen thus producing lactic acid, but no actual evidence exists that *L. crispatus* produces the enzymes to directly degrade glycogen [10, 17]. Lastly, *L. crispatus-*dominated VM are associated with an anti-inflammatory vaginal cytokine profile [18, 19] and immune evasion is likely a crucial (but poorly studied) factor that allows *L. crispatus* to dominate the vaginal niche. A proposed underlying mechanism is that *L. crispatus* produces immunomodulatory molecules [20], but *L. crispatus* may also accomplish immune modulation by alternating its cell surface glycosylation, as has been suggested for gut commensals [21]. Taken together, there is a clear need to study the properties of more human (clinical) *L. crispatus* isolates to fully appreciate the diversity within this species.

Here we investigated whether *L. crispatus* strains isolated from the vaginal tract of women with LVM are pheno- or genotypically distinct from *L. crispatus* strains isolated from vaginal samples with DVM, with the aim of identifying bacterial traits pertaining to a successful domination of lactobacilli of the vaginal mucosa.

## Results

### *Lactobacillus crispatus* strain selection and whole genome sequencing

For this study, 40 nurse-collected vaginal swabs were obtained from the Sexually Transmitted Infections clinic in Amsterdam, The Netherlands, from June to August 2012, as described previously by Dols et al. [4]. In total, 33 *L. crispatus* strains were isolated from these samples (*n* = 16 from LVM samples; *n* = 17 *L. crispatus* strains from DVM samples). Following whole genome sequencing, four contigs (*n* = 3 strains from LVM; *n* = 1 strain from DVM) were discarded as they had less than 50% coverage with other assemblies or with the reference genome (125-2-CHN), suggesting that these isolates belonged to a different *Lactobacillus* species. One contig (from a strain isolated from LVM) aligned to the reference genome, but its genome size was above the expected range, suggestive of contamination with a second strain and was therefore also discarded. The remaining 28 isolates (*n* = 12 LVM and *n* = 16 DVM) were assembled and used for comparative genomics. These genomes have been deposited at DDBJ/ENA/GenBank under the accession numbers NKKQ00000000-NKLR00000000. The versions described in this paper are versions NKKQ01000000-NKLR01000000 (Table 1).

**Table 1:** Overview and properties of 28 *L. crispatus* strains isolated from vaginal swabs with *Lactobacillus-*dominated vaginal microbiota or dysbiotic vaginal microbiota

Strain information		Clinical information vaginal sample				Pan-genome overview				
Accession no.	ID	Group	Nugent score	VM cluster [4]	Urogenital infection	Genome size (Mb)	GC content	No. of core genes	No. of accessory genes	No. of unique genes
NKLQ00000000	RL03	LVM	0	II	None	2.52	36.86	1429	846	12
NKLP00000000	RL05	LVM	0	II	None	2.53	36.39	1429	553	243
NKLO00000000	RL06	LVM	0	II	None	2.16	36.92	1429	481	11
NKLM00000000	RL08	LVM	0	I	None	2.25	36.82	1429	606	43
NKLL00000000	RL09	LVM	0	II	None	2.25	36.83	1429	559	21
NKLK00000000	RL10	LVM	0	I	None	2.15	36.91	1429	612	31
NKLJ00000000	RL11	LVM	0	II	None	2.17	36.90	1429	482	5
NKLF00000000	RL16	LVM	3	II	None	2.56	36.49	1429	855	27
NKKX00000000	RL26	LVM	3	II	None	2.21	36.90	1429	525	103
NKKW00000000	RL27	LVM	3	I	None	2.51	36.84	1429	815	78
NKKU00000000	RL29	LVM	2	II	None	2.20	36.88	1429	501	44
NKKR00000000	RL32	LVM	1	II	CA	2.34	36.97	1429	644	63
NKLR00000000	RL02	DVM	9	III	None	2.22	36.88	1429	528	13
NKLN00000000	RL07	DVM	10	IV	None	2.16	36.94	1429	498	6
NKLI00000000	RL13	DVM	9	V	None	2.19	36.89	1429	488	28
NKLH00000000	RL14	DVM	9	V	None	2.56	36.76	1429	837	63
NKLG00000000	RL15	DVM	8	V	CT	2.27	36.79	1429	593	74
NKLE00000000	RL17	DVM	8	III	None	2.31	37.08	1429	605	250
NKLD00000000	RL19	DVM	8	V	None	2.41	36.93	1429	527	117
NKLC00000000	RL20	DVM	10	III	Candida	2.49	36.47	1429	660	41
NKLB00000000	RL21	DVM	9	V	None	2.49	36.79	1429	807	72
NKLA00000000	RL23	DVM	10	III	None	2.30	36.84	1429	621	1
NKKZ00000000	RL24	DVM	9	III	None	2.37	36.72	1429	682	9
NKKY00000000	RL25	DVM	9	V	None	2.32	36.84	1429	618	16
NKKV00000000	RL28	DVM	10	IV	None	2.17	36.88	1429	489	63
NKKT00000000	RL30	DVM	10	IV	None	2.27	36.76	1429	603	20
NKKS00000000	RL31	DVM	10	IV	CA	2.31	36.93	1429	652	48
NKKQ00000000	RL33	DVM	8	I†	TV	2.37	36.73	1429	631	31

*VM* vaginal microbiota, *LVM Lactobacillus*-dominated VM, *DVM* dysbiotic VM, *CT Chlamydia trachomatis*, *CA Condylomata accuminata*, *TV Trichomonas vaginalis*, VM clusters: I—*L. iners*, II—*L. crispatus*, III—*G. vaginalis*-*Sneathia*, IV—*Sneathia*-Lachnospiraceae, V—*Sneathia*

† This sample clustered together with *L. iners-*dominated samples, but contained many reads belonging to BV-associated bacteria

### *Lactobacillus crispatus* pan-genome

**Fig. 1:**
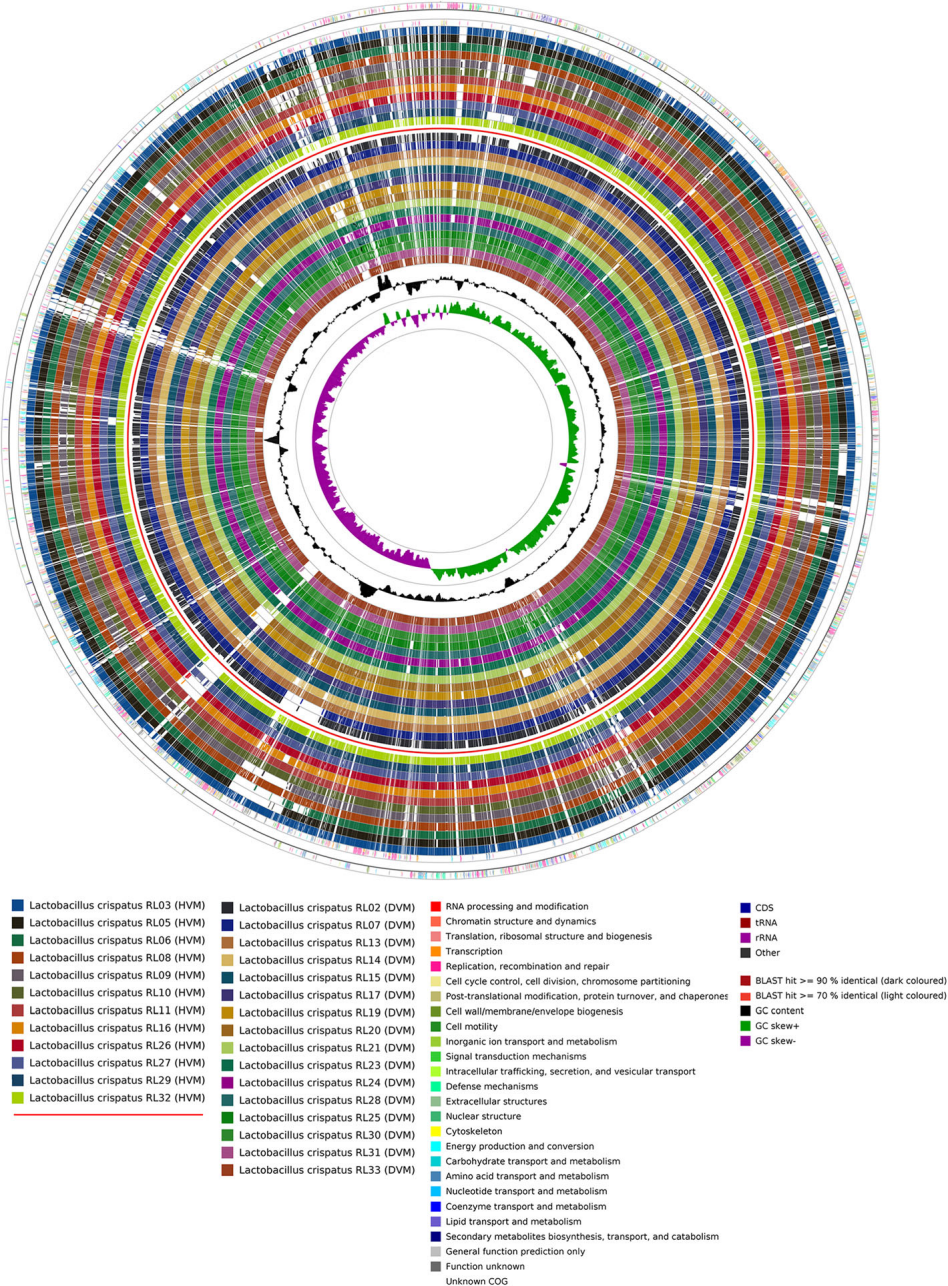
Whole genome alignments of the coding sequences from the *Lactobacillus crispatus* clinical isolates described in this study. The outermost ring represents COG annotated genes on the forward strand (color coded according to the respective COG). The positions of the genes discussed in this article are indicated. The third ring represents COG annotated genes on the reverse strand (color coded according to the respective COG). The next 12 rings each represent one genome of the LVM strains, followed by a separator ring and 16 rings each representing a genome of the DVM strains. The height of the bar and the saturation of the color in these rings indicate a BLAST hit of either > 90% identity (darker colored) or > 70% identity (lightly colored). Hits below 70% identity score are not shown and appear as white bars in the plots. The two inner most rings represent the GC content of that area and the GC-skew respectively. Abbreviations: COG: cluster ortholog genes; LVM: *Lactobacillus*-dominated vaginal microbiota; DVM: dysbiotic vaginal microbiota; WT: wild type

The 28 *L. crispatus* genomes had an average length of 2.31 Mbp (range 2.16–2.56 MB) (Table 1), which was slightly larger than the reference genome (125-2-CHN; 2.04 Mbp). The GC content of the genomes was on average 36.8%, similar to other lactobacilli [10]. An average of 2099 genes were annotated per strain (Table 1; Fig. 1). This set of 28 *L. crispatus* genomes comprised 4261 different gene families. The core genome consisted of 1429 genes (which correspond to ~ 68% of a given genome) and the accessory genome averaged at 618 genes (~ 30%) per strain. Each strain had on average 54 unique genes (~ 2.0%). The number of accessory and unique genes did not significantly differ between strains isolated from LVM or from DVM, with respectively an average of 621 (range 481–855) and 55 (range 5–243) genes for LVM strains and 615 (range 488–837) and 53 (range 1–250) genes for DVM strains. The distribution of cluster of ortholog groups (COG) also did not differ between strains from *Lactobacillus*-dominated and DVM. The gene accumulation model [22] describes the expansion of the pan-genome as a function of the number of genomes assessed and estimated that this species has access to a larger gene pool than described here; the model estimated the *L. crispatus* pan-genome to include 4384 genes.

### A fragmented glycosyltransferase gene was abundant among strains isolated from DVM

In a comparative genomics analysis, we aimed to identify genes that were specific to strains isolated from either LVM or DVM. We observed that three transposases, one of which was further classified as an IS30 family transposase, were more abundant among strains isolated from DVM than among strains from LVM. IS30 transposases are associated with genomic instability and have previously been found to flank genomic deletions in commercial *Lactobacillus rhamnosus* GG probiotic strains [23]. Most notably, we observed that strains from DVM were more likely to carry three gene fragments of a single glycosyltransferase (GT) than strains isolated from LVM. GTs are enzymes that are involved in the transfer of a sugar moiety to a substrate and are thus essential in synthesis of glycoconjugates like exopolysaccharides, glycoproteins, and glycosylated teichoic acids [24, 25].

**Fig. 2:**
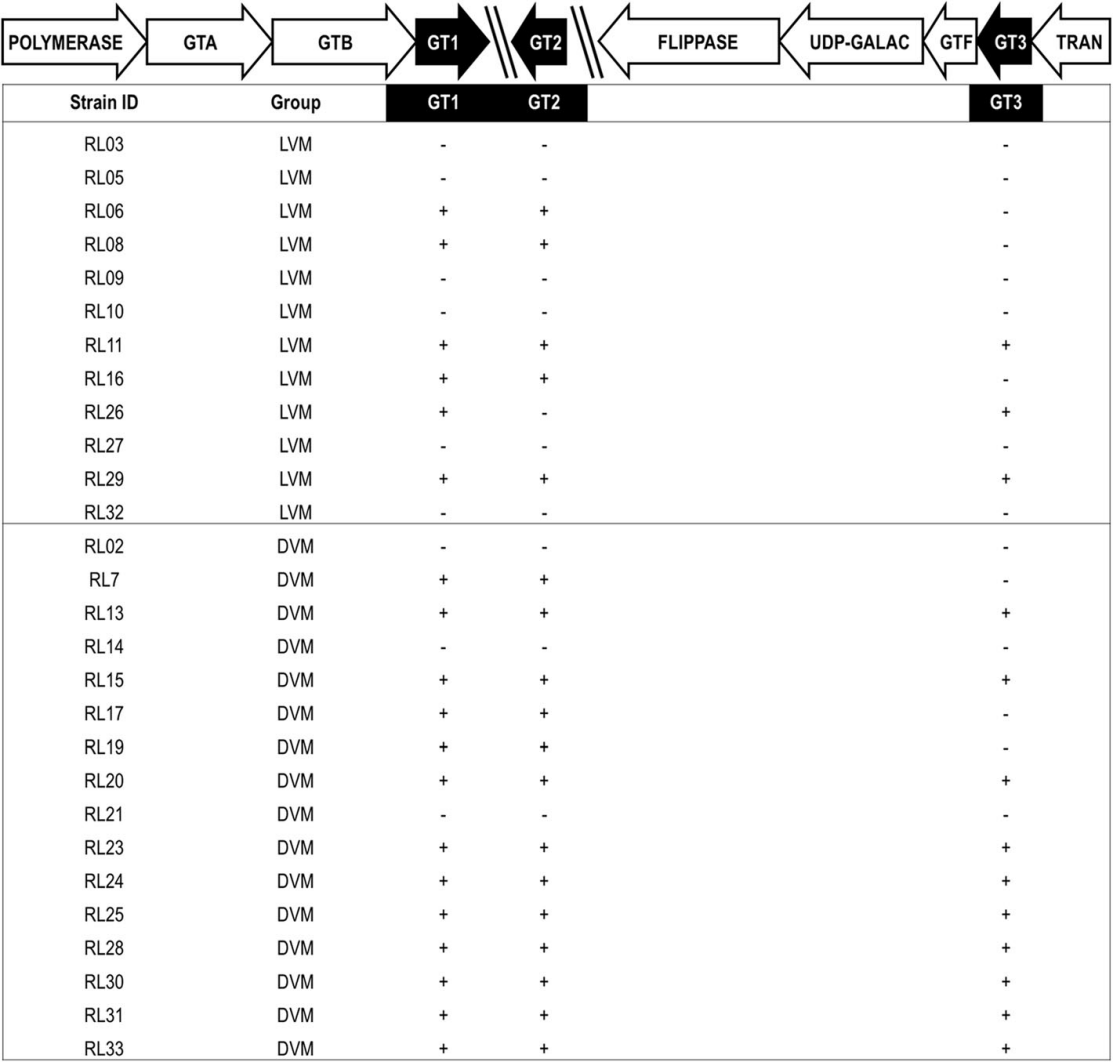
Schematic overview of the organization of the glycosyltransferase fragments in the *Lactobacillus crispatus* genomes. The orientation of the fragments is dependent on the assembly and can therefore be different than depicted here. Also, the distance between the fragments is undetermined and can be of any length (depicted with diagonal lines). Abbreviations: GT: Glycosyltransferase; GTA, GTB: GT super families; GT1, GT2, GT3: GT fragments 1, 2, 3; UDP-GALAC: UDP-Galactopyranose mutase; GTF: GT family 1; TRAN: transposase; LVM: *Lactobacillus*-dominated vaginal microbiota; DVM: dysbiotic vaginal microbiota

**Fig. 3:**
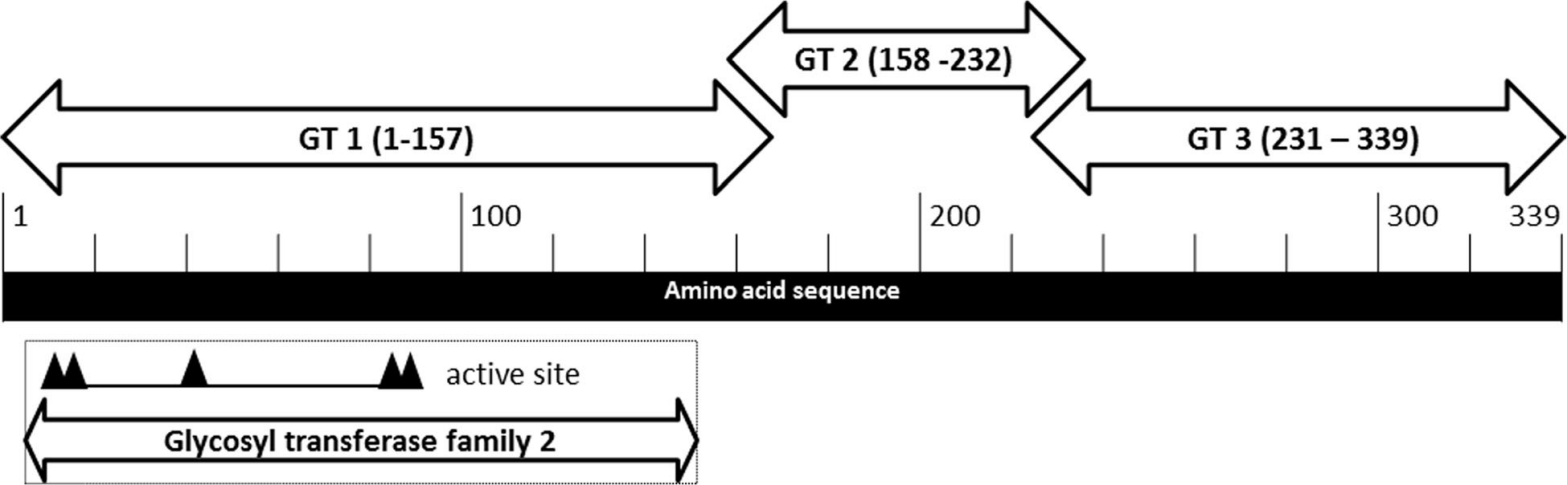
Schematic overview of how the glycosyltransferase fragments align to the *Lactobacillus crispatus* 125-2-CHN reference genome. The first fragment comprises the conserved glycosyltransferase family 2 domain with catalytic activity. The shorter second and third fragments most probably do not harbor any catalytic GT activity. We hypothesize that these two fragments play a role in steering the specific activity of the GT (e.g., towards donor or substrate specificity). Abbreviation: GT: glycosyltransferase

The three differentially abundant GT gene fragments all align to different regions of a single family 2 A-fold GT gene of the 125-2-CHN *L. crispatus* strain (CGA_000165885.1) and are flanked by other genes potentially encoding GTs (Fig. 2). Fragment 1 aligns with 472 bp of the original unfragmented GT, while fragment 2 overlaps with the last 3 bp of fragment 1 and fragment 3 overlaps 7 bp with fragment 2. Given that all these fragments align to the non-fragmented GT gene in *L. crispatus* 125-2-CHN, we hypothesize that the three fragments belong to the same GT. The *L. crispatus* genomes however contained one or more of the three GT fragments, while the surrounding genes were conserved among the strains. The first fragment of 510 bp contains the true GT fold domain and is thus likely responsible for the catalytic activity of the GT. The second and third fragments are considerably shorter, respectively 228 and 328 bp, and do not harbor any significant relation to a known GT fold (Fig. 3). Four different combinations of GT fragments were observed in the studied genomes, namely a variant with (1) no fragments, (2) all three fragments, (3) fragments 1 and 3, and (4) fragments 1 and 2 (Fig. 2; Table 2).

**Table 2:** Comparison of distribution of glycosyltransferase (GT) gene fragments in *Lactobacillus crispatus* genomes isolated from vaginal samples with *Lactobacillus-*dominated or dysbiotic vaginal microbiota

	LVM *N* = 12 (%)	DVM *N* = 16 (%)	*p* value*
No GT fragments	6 (50.0)	3 (18.8)	0.114
1st and 2nd GT fragments	3 (25.0)	3 (18.8)	1.000
1st and 3rd GT fragment	1 (8.3)	0 (0.0)	0.429
All 3 GT fragments	2 (16.6)	10 (62.5)	0.023

*LVM Lactobacillus*-dominated VM, *DVM* dysbiotic VM

*Fisher’s exact test

### Strains isolated from LVM were not phenotypically distinct from strains isolated from DVM

Phenotypic studies on the *L. crispatus* strains did not reveal any biofilm formation—as assessed by crystal violet assays, except for one strain (RL19) which produced a weak biofilm. In line with this, very low levels of autoaggregation (on average 5%) were observed and this also did not differ between the two groups of strains. Strain-specific carbohydrate fermentation profiles were observed, as assessed by a commercial API CH50 test, but the distribution of these profiles did not relate to whether the strains were isolated from LVM or from DVM. Strains isolated from LVM produced similar amounts of organic acids compared with strains isolated from DVM when grown on chemically defined medium mimicking vaginal fluids [26]. The strains mainly produced lactic acid. Other acids such as succinic acid, butyric acid, glutamic acid, phenylalanine, isoleucine, and tyrosine were also produced, but fourfold lower compared to lactic acid. Very small acidic molecules, such as acetic and propionic acid, were out of the detection range and could thus not be measured. We also assessed antimicrobial activity against a common urogenital pathogen *Neisseria gonorrhoeae.* Inhibition was similar for strains isolated from LVM and from DVM: *N. gonorrhoeae* growth was inhibited (i.e., lower OD_600nm_ in stationary phase compared to the control), in a dose-dependent way, by on average 27.9 ± 15.8% for undiluted *L. crispatus* supernatants compared to the *N. gonorrhoeae* control. Undiluted neutralized *L. crispatus* supernatants inhibited *N. gonorrhoeae* growth by on average 15.7 ± 16.3% (Additional file 1).

### Strain-specific glycogen growth among both LVM and DVM isolates

**Fig. 4:**
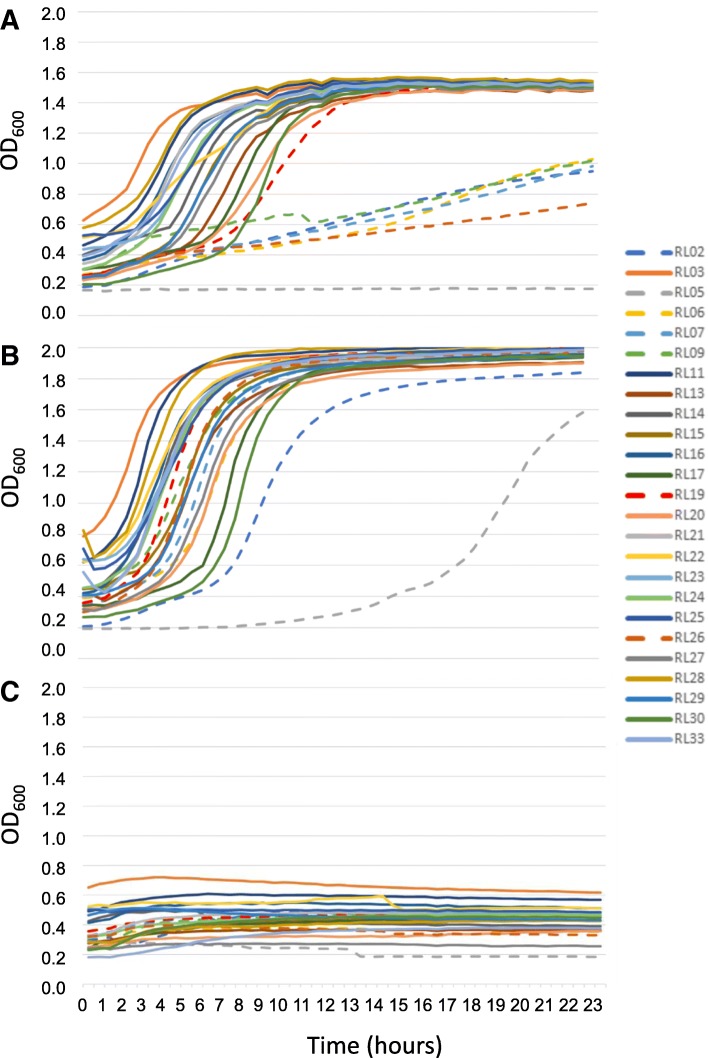
Correlation between growth on glycogen and N-terminal mutation in a putative type I pullulanase protein. Growth curves for individual *Lactobacillus crispatus* strains on minimal medium supplemented with **a** glycogen, **b** glucose, and **c** water. Strains that showed less-efficient or no growth on glycogen carried a mutation in the N-terminal sequence of a putative type I pullulanase protein and are indicated with a dashed line

Of the 28 strains for which full genomes were available, we tested 25 strains (*n* = 12 LVM and *n* = 13 DVM) for growth on glycogen. We compared growth on glucose-free NYCIII medium supplemented with glycogen as carbon source to growth on NYCIII medium supplemented with glucose (positive control) and NYCIII medium supplemented with water (negative control). All except one strain (RL05) showed growth on glycogen; however, six strains showed substantially less-efficient growth on glycogen. One strain showed a longer lag time (RL19; on average 4.5 h, compared to an average of 1.5 h for other strains), and five strains (RL02, RL06, RL07, RL09, and RL26) showed a lower OD after 36 h of growth compared to other strains (Fig. 4). Growth on glycogen did not correlate to whether the strain was isolated from LVM or DVM.

### Growth on glycogen corresponded with variation in a putative pullulanase type I gene

We followed up on the glycogen growth experiments with a gene-trait analysis as glycogen is considered to be a key, although disputed, nutrient (directly) available to *L. crispatus*. We searched the *L. crispatus* genomes for the presence/absence of enzymes that can potentially be involved in glycogen metabolism. We thus searched for orthologs of the (1) glycogen debranching enzyme (encoded by *glgX*) in *Escherichia coli* [27, 28], (2) *Streptococcus agalactiae* pullulanase [29], (3) SusB of *Bacteroides thetaiotaomicron* [30], and (4) the amylase (encoded by *amyE*) of *Bacillus subtilis* [31]. This search revealed a gene that was similar to the *glgX* gene; this gene was annotated as a pullulanase type I gene. In other species, this pullulanase is bound to the outer S-layer of the cell wall, suggesting that this enzyme utilizes extracellular glycogen [32]. All except two strains (RL31, RL32) carried a copy of this gene. The genes are conserved except for variation in the sequence that encodes a putative N-terminal signal peptide that may be involved in extracellular localization of the enzyme. All strains with less-efficient growth on glycogen had a 29-amino acid deletion in the N-terminal sequence (strains: RL02, RL06, RL07, RL09, RL19, and RL26) and the strain that showed no growth (RL05) had an 8-amino acid deletion in the same region as the other strains in addition to 37-amino acid deletion further downstream (Table 3).

**Table 3:** Overview of *Lactobacillus crispatus* strain-specific growth on glycogen and corresponding translated amino acid sequence at the N-terminal of a pullulanase type I gene

Strain ID	Group	Growth on glycogen	Pullulanase type I amino acid sequence (N-terminal)
RL3	LVM	+	MILWRNLFMNKKSGHNIKFKSIFVCTSAIMSLWLGANLTTTQVHAAEDNAAPKSSEVVGQTNSSKDNAATATVQNQSNAKAKQRQQGVAPQNVPTVLAA
RL5	LVM	–	M________NKKSGHNIKFKSIFVCTSAIMSLWLGANLTTTQVHAAEDNAAP_____________________________________PQNVPTVLAA
RL6	LVM	+/−	M_____________________________SLWLGANLTTTQVHAAEDNAAPKSSEVVGQTNSSKDNAATATVQNQSNAKAKQRQQGVAPQNVPTVLAA
RL8	LVM	NA	MILWRNLFMNKKSGHNIKFKSIFVCTSAIMSLWLGANLTTTQVHAAEDNAAPKSSEVVGQTNSSKDNAATATVQNQSNAKAKQRQQGVAPQNVPTVLAA
RL9	LVM	+/−	M_____________________________SLWLGANLTTTQVHAAEDNAAPKSSEVVGQTNSSKDNAATATVQNQSNAKAKQRQQGVAPQNVPTVLAA
RL10	LVM	NA	MILWRNLFMNKKSGHNIKFKSIFVCTSAIMSLWLGANLTTTQVHAAEDNAAPKSSEVVGQTNSSKDNAATATVQNQSNAKAKQRQQGVAPQNVPTVLAA
RL11	LVM	+	MILWRNLFMNKKSGHNIKFKSIFVCTSAIMSLWLGANLTTTQVHAAEDNAAPKSSEVVGQTNSSKDNAATATVQNQSNAKAKQRQQGVAPQNVPTVLAA
RL16	LVM	+	MILWRNLFMNKKSGHNIKFKSIFVCTSAIMSLWLGANLTTTQVHAAEDNAAPKSSEVVGQTNSSKDNAATATVQNQSNAKAKQRQQGVAPQNVPTVLAA
RL22†	LVM	+	MILWRNLFMNKKSGHNIKFKSIFVCTSAIMSLWLGANLTTTQVHAAEDNAAPKSSEVVGQTNSSKDNAATATVQNQSNAKAKQRQQGVAPQNVPTVLAA
RL26	LVM	+/−	M_____________________________SLWLGANLTTTQVHAAEDNAAPKSSEVVGQTNSSKDNAATATVQNQSNAKAKQRQQGVAPQNVPTVLAA
RL27	LVM	+	MILWRNLFMNKKSGHNIKFKSIFVCTSAIMSLWLGANLTTTQVHAAEDNAAPKSSEVVGQTNSSKDNAATATVQNQSNAKAKQRQQGVAPQNVPTVLAA
RL29	LVM	+	MILWRNLFMNKKSGHNIKFKSIFVCTSAIMSLWLGANLTTTQVHAAEDNAAPKSSEVVGQTNSSKDNAATATVQNQSNAKAKQRQQGVAPQNVPTVLAA
RL32	LVM	NC	---------------------------------------------------------------------------------------------------
RL2	DVM	+/−	M_____________________________SLWLGANLTTTQVHAAEDNAAPKSSEVVGQTNSSKDNAATATVQNQSNAKAKQRQQGVAPQNVPTVLAA
RL7	DVM	+/−	M_____________________________SLWLGANLTTTQVHAAEDNAAPKSSEVVGQTNSSKDNAATATVQNQSNAKAKQRQQGVAPQNVPTVLAA
RL13	DVM	+	MILWRNLFMNKKSGHNIKFKSIFVCTSAIMSLWLGANLTTTQVHAAEDNAAPKSSEVVGQTNSSKDNAATATVQNQSNAKAKQRQQGVAPQNVPTVLAA
RL14	DVM	+	MILWRNLFMNKKSGHNIKFKSIFVCTSAIMSLWLGANLTTTQVHAAEDNAAPKSSEVVGQTNSSKDNAATATVQNQSNAKAKQRQQGVAPQNVPTVLAA
RL15	DVM	+	MILWRNLFMNKKSGHNIKFKSIFVCTSAIMSLWLGANLTTTQVHAAEDNAAPKSSEVVGQTNSSKDNAATATVQNQSNAKAKQRQQGVAPQNVPTVLAA
RL17	DVM	+	MILWRNLFMNKKSGHNIKFKSIFVCTSAIMSLWLGANLTTTQVHAAEDNAAPKSSEVVGQTNSSKDNAATATVQNQSNAKAKQRQQGVAPQNVPTVLAA
RL19	DVM	EL	M_____________________________SLWLGANLTTTQVHAAEDNAAPKSSEVVGQTNSSKDNAATATVQNQSNAKAKQRQQGVAPQNVPTVLAA
RL20	DVM	+	MILWRNLFMNKKSGHNIKFKSIFVCTSAIMSLWLGANLTTTQVHAAEDNAAPKSSEVVGQTNSSKDNAATATVQNQSNAKAKQRQQGVAPQNVPTVLAA
RL21	DVM	+	MILWRNLFMNKKSGHNIKFKSIFVCTSAIMSLWLGANLTTTQVHAAEDNAAPKSSEVVGQTNSSKDNAATATVQNQSNAKAKQRQQGVAPQNVPTVLAA
RL23	DVM	+	MILWRNLFMNKKSGHNIKFKSIFVCTSAIMSLWLGANLTTTQVHAAEDNAAPKSSEVVGQTNSSKDNAATATVQNQSNAKAKQRQQGVAPQNVPTVLAA
RL24	DVM	+	MILWRNLFMNKKSGHNIKFKSIFVCTSAIMSLWLGANLTTTQVHAAEDNAAPKSSEVVGQTNSSKDNAATATVQNQSNAKAKQRQQGVAPQNVPTVLAA
RL25	DVM	+	MILWRNLFMNKKSGHNIKFKSIFVCTSAIMSLWLGANLTTTQVHAAEDNAAPKSSEVVGQTNSSKDNAATATVQNQSNAKAKQRQQGVAPQNVPTVLAA
RL28	DVM	+	MILWRNLFMNKKSGHNIKFKSIFVCTSAIMSLWLGANLTTTQVHAAEDNAAPKSSEVVGQTNSSKDNAATATVQNQSNAKAKQRQQGVAPQNVPTVLAA
RL30	DVM	+	MILWRNLFMNKKSGHNIKFKSIFVCTSAIMSLWLGANLTTTQVHAAEDNAAPKSSEVVGQTNSSKDNAATATVQNQSNAKAKQRQQGVAPQNVPTVLAA
RL31	DVM	NC	---------------------------------------------------------------------------------------------------
RL33	DVM	+	MILWRNLFMNKKSGHNIKFKSIFVCTSAIMSLWLGANLTTTQVHAAEDNAAPKSSEVVGQTNSSKDNAATATVQNQSNAKAKQRQQGVAPQNVPTVLAA

*LVM Lactobacillus*-dominated vaginal microbiota, *DVM* dysbiotic vaginal microbiota, *NA* not available, *NC* non-cultivable, *EL* extended lag time

†The genome of RL22 was not deposited in GenBank as the sequencing depth was too low and the N50 and NG50 values gave an inconclusive image of the assembly’s quality

## Discussion

### Key findings of this paper

Here we report the full genomes of 28 *L. crispatus* clinical isolates, the largest contribution of *L. crispatus* clinical isolates to date. These strains were isolated from women with LVM and from women with DVM. A comparative genomics analysis revealed that a glycosyltransferase gene was more frequently found in the genomes of strains isolated from DVM as compared with strains isolated from LVM, suggesting a fitness advantage for carrying this gene in *L. crispatus* under dysbiotic conditions and a role for surface glycoconjugates in microbiota-host interactions. Comparative experiments pertaining to biofilm formation, antimicrobial activity, and nutrient utilization showed that these two groups of strains did not phenotypically differ from each other. Of particular novelty value, we found that these clinical *L. crispatus* isolates were capable of growth on glycogen and that variation in a pullulanase type I gene correlates to the level of this activity.

### Vaginal dysbiotic conditions may pressurize *Lactobacillus crispatus* to vary its glycome

Several studies have shown that vaginal dysbiosis is associated with an increased pro-inflammatory response, including an increase in pro-inflammatory chemokines and cytokines, but also elevated numbers of activated CD4+ T cells [3, 19], although no clinical signs of inflammation are present and vaginal dysbiosis is seen as a condition rather than as a disease [33]. Nonetheless, it indicates that the vaginal niche in a dysbiotic state is indeed under some immune pressure and that immune evasion could be a key (but poorly studied) trait for probiotic bacterial survival and dominance on the vaginal mucosa.

**Fig. 5:**
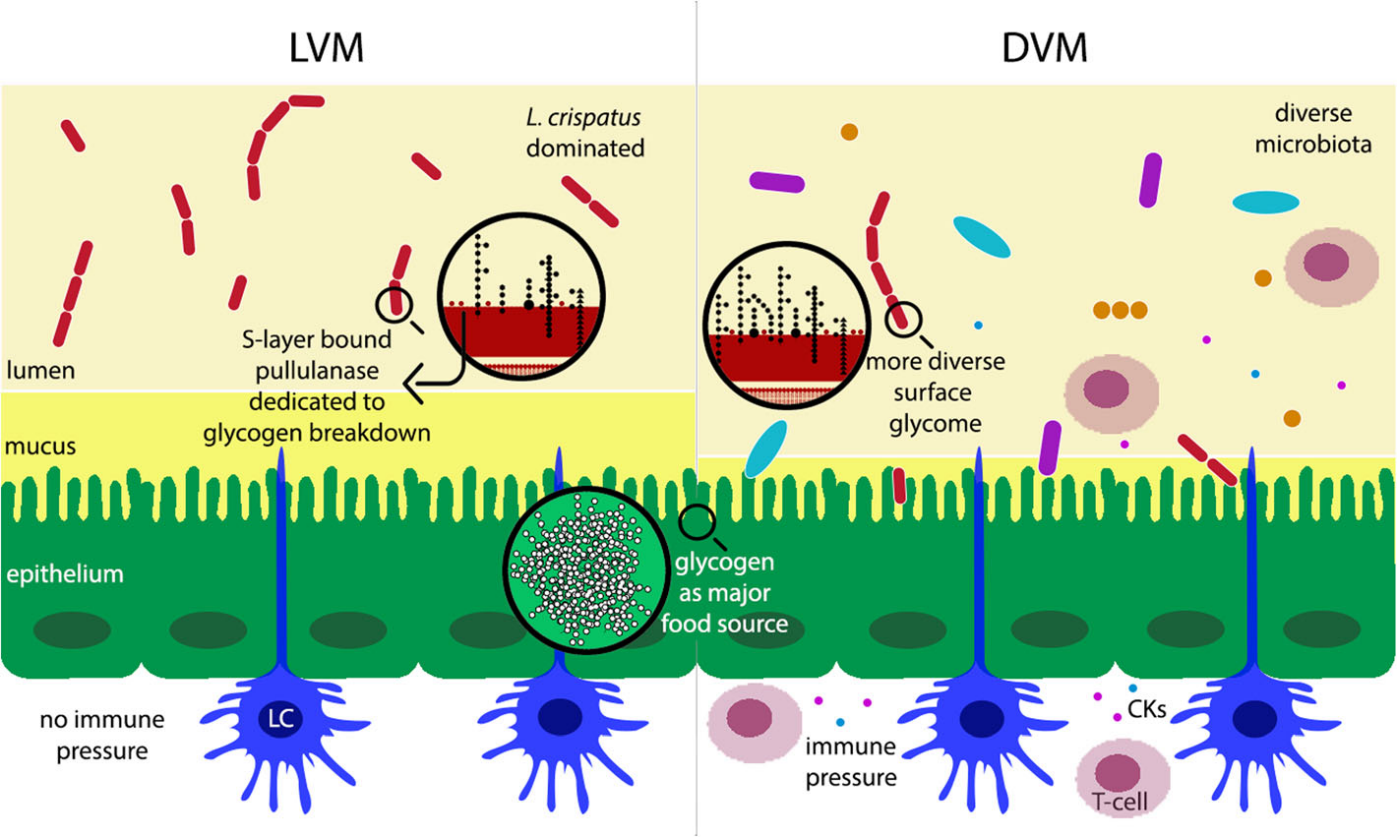
Model for enzymatic activity in glycosylation and glycogen degradation in *Lactobacillus crispatus*. Schematic representation of the vaginal environment with either LVM or DVM. Our comparative genomics analysis revealed a glycosyltransferase gene that was more common in *Lactobacillus crispatus* strains isolated from LVM (red bacteria) and DVM (low abundance of red lactobacilli, diverse bacterial population in multiple colors and forms, thinner mucus layer). We hypothesize that *L. crispatus* in DVM exploits this genetic variation to allow for (a higher) variation in cell wall glycoconjugates providing a mechanism for *L. crispatus* to persist at low levels in DVM and remain stealth from the immune system. Another finding of this work describes the ability of *L. crispatus* strains to utilize glycogen as a food source, which is associated with the presence of a full-length pullulanase gene (red dots on cell wall of *L. crispatus*). Abbreviations: LVM: *Lactobacillus*-dominated vaginal microbiota; DVM: dysbiotic vaginal microbiota, LC: Langerhans cell, CK: cytokines

Our comparative genomics analysis revealed a glycosyltransferase (GT) gene that was more common in strains isolated from DVM compared with strains isolated from LVM. The identified GT consists of three fragments, which all align to a single GT in the reference *L. crispatus* genome (125-2-CHN). Sequence analyses showed that the first and longest fragment exhibits close homology to a known GT-A fold and most probably harbors the active site of the GT (Fig. 3). The latter two fragments do not harbor any structural motifs resembling known GTs and most probably do not harbor any catalytic GT activity. We hypothesize that these two fragments play a role in steering the specific activity of the GT (e.g., towards donor or substrate specificity). This might point towards *L. crispatus* harnessing its genetic potential to change its surface glycome. Such a process is termed phase variation and allows bacteria to rapidly adapt and diversify their surface glycans, resulting in an evolutionary advantage in the arms race between the immune system and invading bacteria. Modulation of the surface glycome by phase variation of the GT coding sequence is a common immune evasion strategy, which has been extensively studied in pathogenic bacteria like *Campylobacter jejuni* [25], but could be utilized by commensals as well [21]. We hypothesize that *L. crispatus* in DVM exploits this genetic variation to allow for (a higher) variation in cell wall glycoconjugates providing a mechanism for *L. crispatus* to persist at low levels in DVM and remain stealth from the immune system (Fig. 5). Of note, evidence for expression of all of the three GT fragments comes from a recent transcriptomics study that studied the effect of metronidazole treatment on the VM of women with (recurring) BV [11]. Personal communication with Dr. Zhi-Luo Deng revealed that high levels of expression for the three putative GT peptides were present in the vaginal samples of two women who were responsive to treatment (i.e., their VM was fully restored to a *L. crispatus*-dominated VM following treatment with metronidazole). See Additional file 2. This finding is in line with our hypothesis that the presence of the fragmented GT gene has a selective advantage for *L. crispatus* under dysbiotic conditions. Further functional experiments are needed to test this hypothesized host-microbe interaction and determine if and how the variation of glycoconjugates is affected by this GT. Additionally, the immunological response of the host must be further studied in reference to these hypothesized microbial adaptations. The bacterial surface glycome and related variability events are currently overlooked features in probiotic strain selection, though they might be crucial to a strain’s survival and in vivo dominance [21].

### No distinct phenotypes pertaining to dominance in vivo were observed

It has previously been postulated, relying merely on genomics data, that the accessory genome of *L. crispatus* could lead to strain differences relating to biofilm formation, adhesion, and competitive exclusion of pathogens [9, 10]; all of which could influence whether a strain dominates the vaginal mucosa or not. Our comparative experimental work, however, showed that *L. crispatus*—irrespective of whether the strain was isolated from a woman with LVM or with DVM—all formed little to no biofilm and demonstrated effective lactic acid production and effective antimicrobial activity against *N. gonorrhoeae.* The previous genomic analyses also suggested that *L. crispatus* has enzymes that can degrade various carbohydrates [10]. Indeed, we observed that *L. crispatus* ferments a broad range of carbohydrates, as assessed by a commercial API test, but these profiles did not differ between strains isolated from LVM or from DVM.

### First evidence showing that *Lactobacillus crispatus* grows on glycogen

**Fig. 6:**
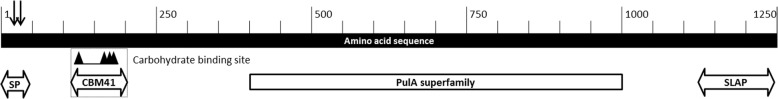
Schematic overview of the organization of the putative pullulanase type I encoding gene in *Lactobacillus crispatus*. The enzyme comprises three conserved domains including an N-terminal carbohydrate-binding module family 41 with specific carbohydrate-binding sites, a catalytic module belonging to the pullulanase super family and a C-terminal bacterial surface layer protein (SLAP). The mutations (indicated by arrows) were located in an unconserved area that encodes a putative signal peptide (SP) that may be involved in subcellular localization. Abbreviations: SP: signal peptide; CBM41: carbohydrate-binding module family 41; PulA: pullulanase; SLAP: surface layer protein

The vaginal environment of healthy reproductive-age women is distinct from other mammals in that it has low microbial diversity, a high abundance of lactobacilli, and high levels of lactic acid and luminal glycogen [34]. It has been postulated that proliferation of vaginal lactobacilli is supported by estrogen-driven glycogen production [35]; however, the “fly in the ointment”—as finely formulated by Nunn et al. [17]—is that evidence for direct utilization of glycogen by vaginal lactobacilli is absent. Moreover, previous reports have stated that the core genome of *L. crispatus* does not contain the necessary genes to break down glycogen [10, 36]. It has even been suggested that *L. crispatus* relies on amylase secretion by the host or other microbes for glycogen breakdown [17, 37], as *L. crispatus* does contain all the appropriate enzymes to consume glycogen breakdown products such as glucose and maltose [36]. Here we provide the first evidence suggesting that *L. crispatus* human isolates are capable of growing on extracellular glycogen and we identified variation in a gene which correlated with this activity. The identified gene putatively encodes a pullulanase type I enzyme belonging to the glycoside hydrolase family 13 [38]. Its closest ortholog is an extracellular cell-attached pullulanase found in *Lactobacillus acidophilus* [32]. The *L. crispatus* pullulanase gene described here carries three conserved domains, comprising an N-terminal carbohydrate-binding module family 41, a catalytic module belonging to the pullulanase super family, and a C-terminal bacterial surface layer protein (SLAP) [39] (Fig. 6). We observed that all except two of the strains in our study carry a copy of this gene. These two strains (RL31 and RL32) were no longer cultivable after their initial isolation. The six strains that showed less-efficient or no growth on glycogen all showed variation in the N-terminal part of the putative pullulanase protein. All of these deletions are upstream of the carbohydrate-binding module in a sequence encoding a putative signal peptide. This may explain the relatively slow and linear growth on glycogen of most of the mutants (Fig. 4a). The enzyme may be synthesized and not secreted in these mutants, but over time as some cells dye and lyse, the enzyme is released into the media allowing slow conversion of glycogen into sugars that can be imported to fuel growth. The presence of a SLAP domain suggests that the enzyme with a functional signal peptide is assigned to the outermost S-layer of the cell wall and is hence expected to be capable of degrading extracellular glycogen [32]. Further experiments are needed to fully characterize this pullulanase enzyme, to demonstrate its ability to metabolize glycogen in vitro, and to assess whether it degrades intra- or extracellular glycogen. Importantly, this pullulanase is likely part of a larger cluster of glycoproteins involved in glycogen metabolism in *L. crispatus*, which should be considered in future research.

Of note, we analyzed just one *L. crispatus* strain per vaginal sample, while it is plausible that multiple strain types co-exist in the vagina. So strain variability in growth on glycogen (and other carbohydrates) might actually benefit the *L. crispatus* population as a whole and explain the variation in growth on glycogen that we observed, especially considering that glycogen availability may fluctuate along with oscillating estrogen levels during the menstrual cycle. When developing probiotics, it could thus be beneficial to select for *L. crispatus* strains that ferment different carbohydrates (in addition to glycogen) [8] and also to supplement the probiotic with a prebiotic [40, 41].

## Conclusion

Here we report whole genome sequences of 28 *L. crispatus* human isolates. Our comparative study led to three novel insights: (1) gene fragments encoding for a glycosyltransferase were disproportionally higher abundant among strains isolated from DVM, suggesting a role for cell surface glycoconjugates that shape vaginal microbiota-host interactions, (2) *L. crispatus* strains isolated from LVM do not differ from those isolated from DVM regarding the phenotypic traits studied here, including biofilm formation, pathogen inhibitory activity, and carbohydrate utilization, and (3) *L. crispatus* is able to grow on glycogen and this correlates with the presence of a full-length pullulanase type I gene.

## Methods

### *L. crispatus* strain selection

For this study, nurse-collected vaginal swabs were obtained from the Sexually Transmitted Infections clinic in Amsterdam, The Netherlands, from June to August 2012, as described previously by Dols et al. [4]. These vaginal samples came from women with LVM (Nugent score 0–3) and from women with DVM (Nugent score 7–10). LVM and DVM vaginal swabs were plated on tryptic soy agar supplemented with 5% sheep serum and 0.25% lactic acid and pH set to 5.5 with acetic acid and incubated under a microaerobic atmosphere (using an Anoxomat; Mart Microbiology B.V., The Netherlands) at 37 °C for 48–72 h. Candidate *Lactobacillus* spp. strains were selected based on colony morphology (white, small, smooth, circular, opaque colonies), and single colonies were subjected to 16S rRNA sequencing. One *L. crispatus* isolate per vaginal sample was taken forward for whole genome sequencing. A DNA library was prepared for these isolates using the Nextera XT DNA Library preparation kit, and the genome was sequenced using the Illumina Miseq generate FASTQ workflow.

### Genome assembly and quality control

All analyses were run on a virtual machine running Ubuntu version 16.02. Contigs were assembled using the Spades assembly pipeline [42]. Contigs were discarded if they had less than 50% coverage with other assemblies or with the reference genome (N50 and NG50 values deviated more than 3 standard deviations from the mean as determined using QUAST [43]). The genomes were assembled with Spades 3.5.0 using default settings. The Spades pipeline integrates read-error correction, iterative k-mer (nucleotide sequences of length k)-based short read assembling, and mismatch correction. The quality of the assemblies was determined with Quast (History 2013) using default settings and the *Lactobacillus crispatus* 125-2-CHN strain as reference genome (Genbank FN692037).

### Genome annotation and comparative genome analysis

After assembly, the generated contigs were sorted with Mauve contig mover [44], using the *L. crispatus* 125-2-CHN strain as reference genome. Contaminating sequences of human origin and adaptor sequences were identified using BLAST and manually removed. The reordered genomes were annotated using the Prokka automated annotation pipeline [45] using default settings. Additionally, the genomes were uploaded to Genbank and annotated using the NCBI integrated Prokaryotic Genome Annotation Pipeline [46]. The annotated genomes were analyzed using the sequence element enrichment analysis (SEER), which looks for an association between enriched k-mers and a certain phenotype [47]. Following the developer’s instructions, the genomes were split into k-mers using fsm-lite on standard settings and a minimum k-mer frequency of 2 and a maximum frequency of 28. The usage of k-mers enables the software to look for both SNPs as well as gene variation at the same time. After k-mer counting, the resulting file was split into 16 equal parts and g-zipped for parallelization purposes. In order to correct for the clonal population structure of bacteria, the population structure was estimated using Mash with default settings [48]. Using SEER, we looked for k-mers of various lengths that associated with whether the *L. crispatus* strains came from LVM or DVM. The results were filtered for k-mers with a chi-square test of association of < 0.01 and a likelihood-ratio test *p* value (a statistical test for the goodness of fit for two models) of < 0.0001. The resulting list of k-mers was sorted by likelihood-ratio *p*, and the top 50 hits were manually evaluated using BLASTx and BLASTn.

### Pan and accessory genome analysis

We used the bacterial pan-genome analysis tool developed by Chaudhari et al. [49] using default settings. The circular image was created using CGview Comparison Tool [50] by running the build_blast_atlas_all_vs_all.sh script included in the package.

### Comparative phenotype experiments

Not all strains were (consistently) cultivable after their initial isolation, so experimental data was collected for a subset of the strains and could differ per experiment. The ratio of cultivable LVM and DVM strains was however similar for each experiment. For a full overview of experimental procedures, we refer to the Additional file 1. In short, carbohydrate metabolism profiles were assessed using commercial API CH50 carbohydrate fermentation tests (bioMérieux, Inc., Marcy l’Etoile, France) according to the manufacturer’s protocol. To assess organic acid production, strains were grown on medium that mimicked vaginal secretions [26]. Total metabolite extracts from spent medium were assessed as previously described by Collins et al. [41]. Biofilm formation was assessed using the crystal violet assay as described by Santos et al. [51] and autoaggregation as described by Younes et al. [52]. Antimicrobial activity against *Neisseria gonorrhoeae* was assessed by challenging *N. gonorrhoeae* (WHO-L strain) with varying (neutralized with NaOH to pH 7.0) dilutions of *L. crispatus* supernatants. Inhibitory effect was assessed as percentile difference in OD_600nm_ in a conditional stationary phase as compared to the control.

### Glycogen degradation assay

Starter cultures were grown in regular NYCIII glucose medium for 72 h. For this assay, 1.1× carbohydrate-deprived NYCIII medium was supplemented with water (negative control), 5% glucose (positive control), or 5% glycogen (Sigma-Aldrich, Saint Louis, USA) and subsequently inoculated with 10% (*v*/*v*) bacterial culture (OD ~ 0.5; 10^9^ CFU/ml). Growth on glycogen was compared to growth on NYCII without supplemented carbon source and to NYCIII with glucose. Growth curves were followed in a BioScreen (Labsystems, Helsinki, Finland). At least two independent experiments per strain were performed in triplicate.

## Additional files

Additional file 1: Biofilm formation, auto-aggregation, carbohydrate degradation, organic acid production, and antimicrobial activity of *Lactobacillus crispatus* strains. (DOCX 2146 kb)

Additional file 2: Transcripts of *Lactobacillus crispatus* glycosyltransferase gene fragments in vaginal samples of women treated for bacterial vaginosis. (XLSX 11 kb)

## Abbreviations

COG: Cluster ortholog groups
DVM: Dysbiotic vaginal microbiota
GT: Glycosyltransferase
LVM: *Lactobacillus*-dominated vaginal microbiota
VM: Vaginal microbiota
