# Supplementary material for: Comparative genomics of human Lactobacillus crispatus isolates reveals genes for glycosylation and glycogen degradation: implications for in vivo dominance of the vaginal microbiota
Source: Microbiome. 2019 Mar 29;7:49. doi: 10.1186/s40168-019-0667-9 (PMC6441167; doi:10.1186/s40168-019-0667-9)
Supplement: Supplementary file 1 — Biofilm formation, auto-aggregation, carbohydrate degradation, organic acid production, and antimicrobial activity of Lactobacillus crispatus strains. (DOCX 2146 kb) [file 40168_2019_667_MOESM1_ESM.docx]

**SUPPLEMENTARY INFORMATION**

**Comparative genomics of human *Lactobacillus crispatus* isolates reveals genes for glycogen degradation and glycosylation; implications for dominance *in vivo* dominance of the vaginal microbiota**

**Summary:** *Lactobacillus crispatus* strains isolated from *Lactobacillus-*dominated vaginal microbiota did not phenotypically differ from *Lactobacillus crispatus* strains isolated from dysbiotic vaginal microbiota**.**

**Background**

A vaginal microbiota dominated by lactobacilli (especially *Lactobacillus crispatus*) is considered a hallmark of vaginal health. Implementing *L. crispatus* as a therapeutic therefore has much potential, but appropriate probiotic strains still need to be identified. Phenotypic properties such as biofilm formation, broad-range carbohydrate metabolism, efficient organic acid production and pathogen inhibition could make some strains ‘fitter’ than others and increase their chances to dominate over other species in the vaginal niche. Here we compared *L. crispatus* strains that had been isolated from *Lactobacillus-*dominated vaginal microbiota (LVM) to *L. crispatus* strains isolated from dysbiotic vaginal microbiota (DVM).

**Results**

*Strain selection*

In total, 33 *L. crispatus* strains (n=16 isolated from LVM; n=17 isolated from DVM) were used for comparative phenotypic experiments. Not all strains were (consistently) cultivable after their initial isolation, so experimental data was collected for a subset of the strains and could differ per experiment. The ratio of cultivable LVM and DVM strains was however similar for each experiment.

*Selected Lactobacillus crispatus growth conditions*

*L. crispatus* strains were cultured in Tryptic Soy broth/agar (TSB) (set to pH 5.5 with CH_3_COOH and supplemented with 5% sheep serum and 0.25% lactic acid) rather than the commonly used *Lactobacillus* culture medium, MRS, as we observed that not all strains were able to grow on MRS broth/agar. Cultures were grown at 37°C under micro aerobic conditions (6% oxygen) and colonies/turbidity usually became visible after 48-72 hours. Two strains (RL31; isolated from LVM and RL32; isolated from DVM) were non-cultivable in TSB or MRS and therefore not used for further experiments. These two strains lacked the putative pullulanase gene (see main text).

*All L. crispatus strains showed low levels of biofilm formation and auto aggregation*

We assessed whether biofilm formation differed between strains isolated from LVM and DVM (n=14 LVM; n=12 DVM). Almost all of these strains did not produce a biofilm. One strain (RL019), isolated from DVM, produced a weak biofilm. Supporting these findings, we observed that auto aggregation was very low (on average 5%) and did not differ between the two groups of strains. See Supplementary Table 1.

*All L. crispatus strains degraded a broad range of carbohydrates*

Enzymatic activity for 48 different substrates was assessed for 21 strains (LVM: n=10; DVM: n=11). Strain specific carbohydrate fermentation profiles were observed, but the distribution of these profiles did not relate to whether the strains were isolated from LVM or from DVM (Supplementary Figure 1A). Specifically, all of these strains fermented glucose and ferric citrate, while most of these strains fermented starch (n=19/21), fructose (n=20/21), maltose (n=20/21), sucrose (n=18/21), glucosamine (n=20/21) and lactose (n=15/21). See supplementary Figure 1A. We observed a discrepancy between the API test results and the glycogen growth experiments; the API test showed that fewer strains fermented glycogen than was found in our growth experiments. However, the API test depends on a color change as indication of growth which may be a less sensitive method to detect growth compared with our glycogen growth experiments. Individual averaged growth curves per strain under the different conditions, i.e. glycogen, glucose and water are shown in Supplementary figure 2. Furthermore, glycogen growth experiments on these strains have since been replicated in a different laboratory and very similar results were obtained; these results are accessible on an ‘open kitchen science log’ (see http://www.reblab.org/ongoing-experiments-log/).

*All L. crispatus strains were proficient at producing organic acids*

We performed a comparative metabolomics analysis for 19 *L. crispatus* strains (n=11 LVM, n=8 DVM) to detect any differentially produced organic acids when the strains were grown on chemically defined medium resembling vaginal fluids*.* The strains mainly produced lactic acid. Other acids such as succinic acid, butyric acid, glutamic acid, phenylalanine, isoleucine and tyrosine were also produced but in four-fold lower amounts than lactic acid. Strains isolated from LVM and DVM produced similar amounts of organic acids. See supplementary Figure 1B.

*L. crispatus strains did not differ in antimicrobial activity*

We assessed whether *L. crispatus* strains (LVM: n=11; and DVM: n=8) exhibited any inhibitory effect against each other – as a proxy for strain specific bacteriocin production, but none of the strains inhibited the growth of another strain (data not shown). We studied the inhibitory effect against *N. gonorrhoeae* of four *L. crispatus* strains (LVM: n=2; DVM: n=2). *N. gonorrhoeae* growth was inhibited (i.e. lower OD in stationary phase compared to the control), in a dose-dependent way, by on average 27.9 ± 15.8% for undiluted *L. crispatus* supernatants. Undiluted neutralized *L. crispatus* supernatants inhibited *N. gonorrhoeae* growth by on average 15.7 ± 16.3%, suggesting a pH dependent effect. However, no differences between *L. crispatus* strains coming from LVM or DVM were observed*.* See supplementary Figure 3.

**Methods**

*Biofilm and auto-aggregation*

Biofilm formation was assessed using the crystal violet assay as described by Santos *et al.* [1]. Briefly, medium was inoculated with 10% (v/v) bacterial broth (OD~0.5; 10^9^ CFU/ml) and cultured at 37°C in 6% O_2_ for 72 hours in untreated 96 wells polystyrene micro plates. Wells were rinsed with demineralized water, fixated with 96% ethanol and dyed with 3% crystal violet. Bound crystal violet was dissolved in acetic acid (33%) and the OD_580nm_ was measured. Medium acted as negative control and *Pseudomonas aeroginosa* (ATCC 70888) as positive control. Three independent experiments were performed in triplicate. Autoaggregation was assessed using the assay described by Younes *et al.* 2012. Briefly, cell suspensions containing 10^9^ CFU/ml were spun down, washed twice with PBS and dissolved in 2ml PBS. After brief vortexing, the OD was measured and the suspensions were left stationary at room temperature. The OD was measured again after 4 hours. Autoaggregation was calculated according to the following formula: Autoaggregation: % = (ODt=0 - ODt =4)/ODt=0 x 100. Two independent experiments were performed in duplicate.

*Carbohydrate metabolism*

Carbohydrate degradation profiles were assessed using API CH50 carbohydrate fermentation tests (bioMérieux, Inc., Marcy l'Etoile, France) according to the manufacturer’s protocol. In brief, the lactobacilli were cultivated on TSA plates for 72 hours. API 50 CHL medium (bioMérieux, Inc., Marcy l'Etoile, France) was inoculated with several identical colonies to obtain a suspension with turbidity equivalent to 2 MacFarland (OD_600nm_ ~0.5). The suspension was thoroughly mixed and added to each capsule of the fermentation strips. All capsules were covered with mineral oil (to ensure anaerobic conditions) and the strips were incubated at 37°C for 72 hours. The strips were analyzed for colour changes after 72 hours. Each capsule has a different carbohydrate substrate and the API 50 CHL medium contains bromescal purple, a colour indicator that turns yellow at pH levels lower than 5, i.e. when fermentation has taken place.

*Organic acids produced under vaginal niche conditions*

*L. crispatus* strains were grown in Vaginally Defined Media + Peptone (VDMP; Geshnizgani *et al.* [2]) that mimicked vaginal secretions. Total metabolites were extracted from spent medium. Extracts were separated using high performance liquid chromatography and metabolites were detected with a hydrophilic interaction column as previously described [3]. This method was chosen as it is most suitable for identifying highly polar metabolites, such as organic acids. In brief, cell suspensions were spun down and 50% methanol was added a 1:1 v/v ratio for 30 minutes at 4°C. Samples were then passed through 0.45 μm PTFE syringe filter (Whatman) into amber glass HPLC vials. Samples were vortexed for 15 sec, then transferred to micro-inserts and directly injected into an Agilent 1290 Infinity HPLC coupled to a Q-Exactive Orbitrap mass spectrometer (Thermo-Fisher) with a HESI (heated electrospray ionization) source. Full MS scanning between the ranges of m/z 50-750 was performed on all samples in negative and positive mode. Raw data files were converted to .MZML format and centroid using ProteoWizard [4]. Further data analysis was performed in R using the XCMS package [5] to denoise the data and identify true peaks. Areas under the peak were log base 2 transformed for further statistical analyses. Outcomes were grouped by whether the strains came from LVM or DVM and mass/ionization ratios that differed in intensity (i.e. amount of metabolite that was detected) between the two groups at a statistically significant level after false discovery rate correction were searched in the METLIN metabolite database for further identification.

*Inhibitory effect against same species strains (indication of bacteriocin production)*

To assess whether any of the *L. crispatus* strains inhibited the growth of the other strains we performed a diffusion assay [6]. Holes were cut into Tryptic Soy Agar plates using sterilized utensils and filled with equimolar amounts of filtered supernatants from stationary phase *L. crispatus* cultures. Each strain was plated separately on plates containing supernatants from all the other strains. The plates were cultured microaerobically at 37ºC and were checked for zones of inhibition after 72 hours.

*Inhibitory effect against the urogenital pathogen, Neisseria gonorrhoeae*

We assessed antimicrobial activity of *L. crispatus* supernatants against the *N. gonorrhoeae* WHO-L strain. *N. gonorrhoeae* was grown in TSB supplemented with 5% serum, pH 7, in 5% CO2 atmosphere at 37°C. Stationary phase *L. crispatus* supernatants were sterilized through a 0.45 µm filter and added in a 1:1 ratio as undiluted, 10x or 100x diluted to the *N. gonorrhoeae* culture (10% inoculate with OD~0.5 = 10^8^ CFUs/ml; 10^7^ bacteria per experimental condition). To circumvent the effect of glucose depletion, *N. gonorrhoeae* cultures were inoculated in 2x concentrated TSB, pH 7. To evaluate a pH dependent effect, the *L. crispatus* supernatant dilutions were aliquotted at the start of each experiment and one aliquot per dilution was neutralized with NaOH. Sterile demi water acted as negative control. To evaluate the effect of pH alone, *N. gonorrhoeae* cultures were also inoculated in TSB set to pH5.5. Growth curves were followed in a BioSCreen (Labsystems, Helsinki, Finland). All conditions were included in triplicate and performed in two independent experiments. Inhibitory effect was assessed as difference in OD_600nm_ in a conditional stationary phase as compared to the control.

**Supplementary table 1.** Overview of auto-aggregation and biofilm formation per *Lactobacillus crispatus* strain isolated from either LVM or DVM.

| **Strain ID** | **Group** | **Auto-aggregation (%)** | **Biofilm formation** |
| --- | --- | --- | --- |
| RL1 | LVM | NA | None |
| RL3 | LVM | NA | None |
| RL4 | LVM | 0.0 | None |
| RL5 | LVM | 0.0 | None |
| RL6 | LVM | 0.0 | None |
| RL8 | LVM | NA | None |
| RL9 | LVM | 8.5 | None |
| RL10 | LVM | 14.4 | None |
| RL11 | LVM | 5.0 | None |
| RL12 | LVM | 0.0 | None |
| RL16 | LVM | 6.8 | None |
| RL22 | LVM | 0.0 | None |
| RL26 | LVM | 12.5 | None |
| RL27 | LVM | 4.1 | None |
| RL29 | LVM | 14.1 | NA |
| RL32 | LVM | NA | NA |
| RL2 | DVM | 6.6 | None |
| RL7 | DVM | 5.2 | None |
| RL13 | DVM | NA | NA |
| RL14 | DVM | NA | NA |
| RL15 | DVM | 4.6 | None |
| RL17 | DVM | NA | NA |
| RL18 | DVM | NA | NA |
| RL19 | DVM | 0.0 | Weak |
| RL20 | DVM | 7.1 | None |
| RL21 | DVM | 0.0 | None |
| RL23 | DVM | 13.0 | None |
| RL24 | DVM | 0.0 | None |
| RL25 | DVM | 0.0 | None |
| RL28 | DVM | 0.0 | None |
| RL30 | DVM | NA | None |
| RL31 | DVM | NA | NA |
| RL33 | DVM | 13.1 | None |

LVM: *Lactobacillus*-dominated vaginal microbiota; DVM: dysbiotic vaginal microbiota; NA: not available; None: Non-biofilm producer (OD ≤ ODc); Weak: Weak biofilm producer (ODc < OD ≤ 2 x ODc)

**Supplementary figure 1.** Fermentation profiles were similar for strains isolated from LVM and DVM: A) Percentage of *Lactobacillus crispatus* strains that fermented the substrates of a commercial API CH 50 test (bioMérieux, Inc., Marcy l'Etoile, France); B) Averaged organic acid production per group when strains were grown in chemically defined medium mimicking vaginal fluids. Abbreviations: LVM: *Lactobacillus*-dominated vaginal microbiota; DVM: dysbiotic vaginal microbiota.


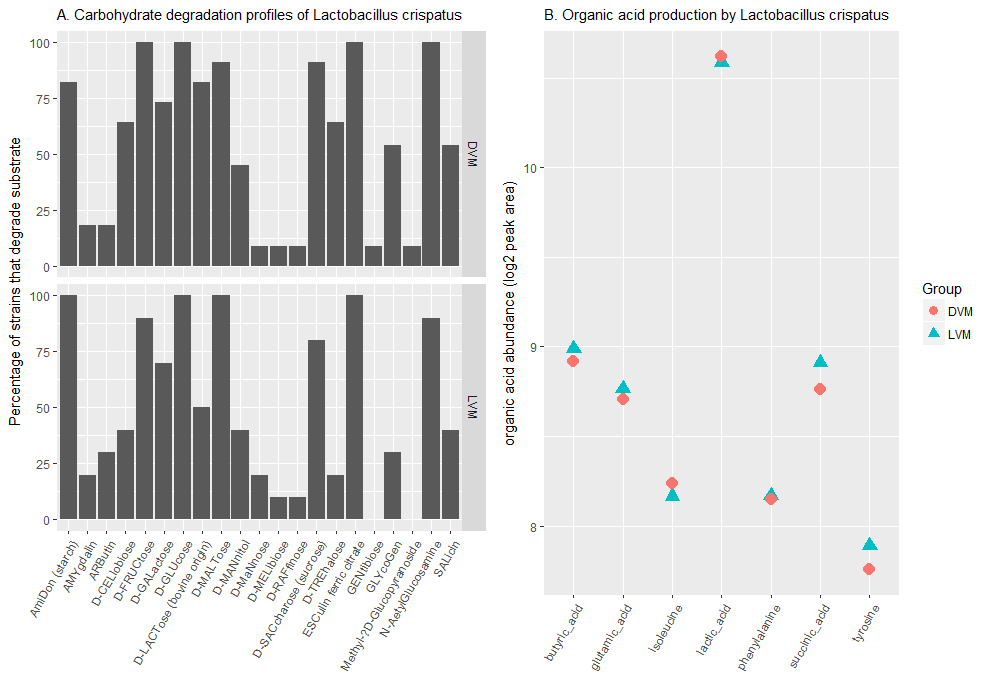


**Supplementary figure 2.** Inhibition of *Neisseria gonorrhoeae* (WHO-L strain) growth by *Lactobacillus crispatus* was similar for A) LVM and B) DVM strains. *N. gonorrhoeae* cultures were challenged with sterilized (un)diluted, (un)neutralized *L. crispatus* supernatants. Averaged results for LVM and DVM strains are shown. Abbreviations: LVM: *Lactobacillus*-dominated vaginal microbiota; DVM: dysbiotic vaginal microbiota; LC: *Lactobacillus crispatus*, TSB: Tryptic Soya Broth


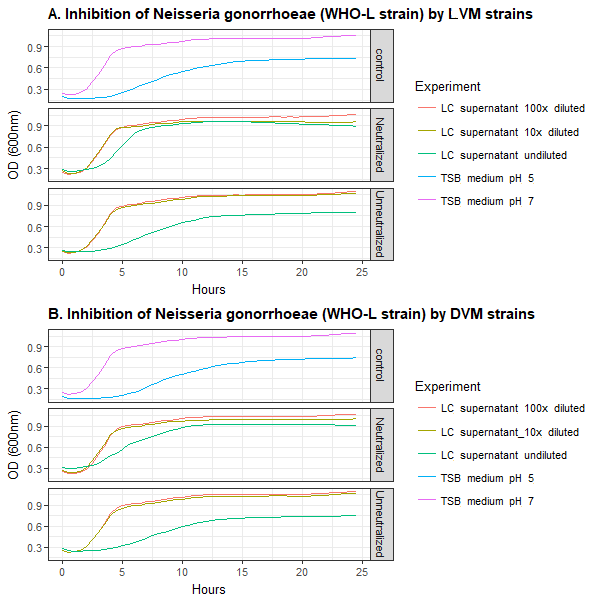


**REFERENCES**

1. Santos CM, Pires MC, Leao TL, Hernandez ZP, Rodriguez ML, Martins AK, Miranda LS, Martins FS, Nicoli JR: **Selection of *Lactobacillus* strains as potential probiotics for vaginitis treatment**. *Microbiology* 2016, **162**(7):1195-1207.

2. Geshnizgani AM, Onderdonk AB: **Defined medium simulating genital tract secretions for growth of vaginal microflora**. *J Clin Microbiol* 1992, **30**(5):1323-1326.

3. Collins SL, McMillan A, Seney S, van der Veer C, Kort R, Sumarah MW, Reid G: **Promising Prebiotic Candidate Established by Evaluation of Lactitol, Lactulose, Raffinose, and Oligofructose for Maintenance of a *Lactobacillus*-Dominated Vaginal Microbiota**. *Appl Environ Microbiol* 2018, **84**(5).

4. Kessner D, Chambers M, Burke R, Agus D, Mallick P: **ProteoWizard: open source software for rapid proteomics tools development**. *Bioinformatics* 2008, **24**(21):2534-2536.

5. Mahieu NG, Genenbacher JL, Patti GJ: **A roadmap for the XCMS family of software solutions in metabolomics**. *Curr Opin Chem Biol* 2016, **30**:87-93.

6. Martin R, Soberon N, Vaneechoutte M, Florez AB, Vazquez F, Suarez JE: **Characterization of indigenous vaginal lactobacilli from healthy women as probiotic candidates**. *Int Microbiol* 2008, **11**(4):261-266.
